# Development and Validation of Echocardiography Artificial Intelligence Models: A Narrative Review

**DOI:** 10.3390/jcm14197066

**Published:** 2025-10-07

**Authors:** Sadie Bennett, Casey L. Johnson, George Fisher, Fiona Erskine, Samuel Krasner, Andrew J. Fletcher, Paul Leeson

**Affiliations:** 1 Oxford Cardiovascular Clinical Research Facility, Division of Cardiovascular Medicine, Radcliffe Department of Medicine, University of Oxford, Oxford OX3 9DU, UK; 2 Heart & Lung Clinic, University Hospitals of North Midlands NHS Trust, Stoke-on-Trent ST4 6QG, UK; 3 Department of Cardiology, King’s College Hospital, London SE5 9RS, UK; 4 John Radcliffe Hospital, Oxford University Hospitals NHS Foundation Trust, Oxford OX3 9DU, UK; 5 Department of Cardiology, Royal Papworth Hospital NHS Foundation Trust, Cambridge CB2 0AY, UK

**Keywords:** artificial intelligence, echocardiography, supervised learning, unsupervised learning, development, validation

## Abstract

Echocardiography is a first-line, non-invasive imaging modality widely used to assess cardiac structure and function; however, its interpretation remains highly operator dependent and subject to variability. The integration of artificial intelligence (AI) into echocardiographic practice holds the potential to transform workflows, enhance efficiency, and improve the consistency of assessments across diverse clinical settings. Interest in the application of AI to echocardiography has grown significantly since the early 2000s with AI models that assist with image acquisition, disease detection, measurement automation, and prognostic stratification for various cardiac conditions. Despite this momentum, the safe and effective deployment of AI models relies on rigorous development and validation practices, yet these are infrequently described in the literature. This narrative review aims to provide a comprehensive overview of the essential steps in the development and validation of AI models for echocardiography. Additionally, it explores current challenges and outlines future directions for the integration of AI within echocardiography.

## 1. Introduction

Echocardiography is a widely available, relatively low-cost, and often first-choice imaging modality that enables real-time assessment of the heart’s structure and function [1]. It therefore plays a crucial role in the diagnosis, treatment, and management of a broad spectrum of cardiovascular conditions [2]. However, echocardiography requires a high level of operator experience, often involving complex analytical workflows and subjective interpretation of acquired images [3]. These factors can contribute to diagnostic errors and variability [4], potentially hindering patient care.

Artificial intelligence (AI) is a broad overarching term that covers any computer programme (algorithms and models) that mimics human intelligence [4]. Within AI, several subfields exist including machine learning, deep learning, natural language processing, and representation learning. The most common sub-field of AI that is used in echocardiography is machine learning. In this context, algorithms are developed that enable models to learn from provided data, allowing them to make predictions or decisions based on predefined objectives [5]. Despite the inception of AI occurring in the 1950s, the application of AI within echocardiography has only recently seen a surge in interest. This began in the early 2000s with an exponential increase in the number of research studies since 2018 [3].

The application of AI within echocardiography offers a promising opportunity to address the known limitations of echocardiography. Through the assessment of tabular data, echocardiography images, or a combination of both, AI aims to alleviate workload burden and increase diagnostic accuracy [6]. To date, AI has demonstrated potential in supporting image acquisition [7,8], disease detection [9,10,11], and measurement automation [12,13,14]. Beyond improving efficiency and accuracy, integrating AI into echocardiography may also enable the development of personalised patient care pathways, tailoring diagnostic and treatment strategies to the individual [15].

Despite significant progress in applying AI to echocardiography, the safe and effective deployment of these technologies depends on rigorous model development and validation [16]. However, these critical concepts are often underrepresented within the literature. While several prior reviews have focused on the applications of AI models within echocardiography [15,17,18,19], this narrative review aims to provide healthcare professionals with a practical and comprehensive understanding of how such models are developed, validated, and implemented into clinical practice. To provide a broad representation of the available literature, we conducted a non-systematic literature search using PubMed, Embase, and Web of Science to identify relevant studies published between 2015 and 2025. These dates were selected to capture the most recent developments within this area. Search terms included ‘artificial intelligence’, ‘machine learning’, ‘echocardiography’, ‘model development’, and ‘model validation’. Additional articles were identified through citation checking and consultation with experts within this area. Studies were selected based on relevance to the development and validation of AI models in echocardiography.

## 2. Development of AI Models Within Echocardiography

**Figure 1:**
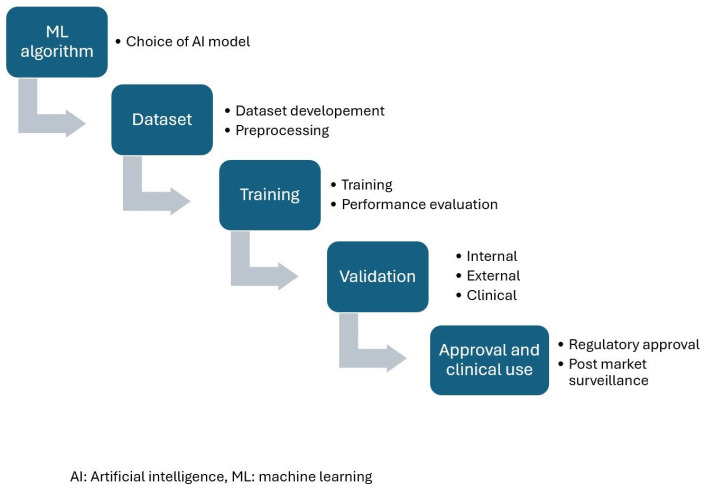
Steps in AI model development and validation.

The development of AI models for echocardiography typically involves several distinct phrases. These steps are shown in Figure 1 and described below.

### 2.1. Choice of Echocardiography Artificial Intelligence Model

There are now numerous technical approaches that can be adopted to develop AI models for use within echocardiography and model development requires in-depth experience of computational approaches. Most models are therefore best developed collaboratively between clinical staff and computational scientists or biomedical engineers. Machine learning techniques are commonly employed when developing an echocardiography AI solution. Briefly, these models are presented with data, often vast and diverse in nature; they then ‘learn’ how to analyse or comprehend this data to support a particular task. This enables models to make predictions, decisions, or take actions without being explicitly programmed [4,20]. There are several broad approaches to machine learning including supervised learning, unsupervised learning, reinforcement learning, and foundational models. When considering which machine learning approach to use, their strengths and weaknesses should be considered and these have been described in detail previously [17,20]. Ultimately, the most appropriate machine learning approach will depend on the research objective and the availability of echocardiography images or tabular data.

In supervised learning algorithms, an AI model is trained using labelled input–output pairs, where each input is associated with a known, correct output. The objective is for the model to learn the underlying mapping between inputs and outputs, enabling it to accurately predict or classify previously unseen data [18]. Supervised learning approaches may be desired if classification is the primary aim with examples including sorting patients into differing disease states. In this approach, input data is labelled or coded prior to training and outputs are compared to a defined ground truth. The ground truth is often a widely accepted gold standard or expert consensus indicating the best possible ‘correct’ value for a given task [3]. Alternatively, in unsupervised learning algorithms, the model is provided with unlabelled data (input or output). The goal here is for the model to analyse the underlying structure of the data and discover hidden patterns, groupings, or relationships without explicit guidance [18]. Unsupervised methods may be useful in identifying previously unknown phenotypes of a specific cardiovascular condition, or patterns that are not immediately recognisable within a dataset [3]. With most model developments, multiple different steps are required that may combine different machine learning approaches. For example, there may be a supervised approach to extract information from images combined with methodologies to link the extracted information with raw image data. In addition, nowadays each single step may combine different machine learning techniques to refine the model’s accuracy.

### 2.2. Echocardiography Datasets

A robust dataset for AI model development in echocardiography may include patient demographics, tabular or images obtained from the echocardiogram alongside biochemistry markers, or medical imaging from alterative imaging modalities.

In order to develop accurate, reproducible, and clinically useful AI models, it is crucial that datasets used in model development be of sufficient quality and size to accurately reflect the intended use population [15]. If data is limited, erroneous, or missing, the resulting AI model risks producing inaccurate or biassed outputs, which can negatively affect their predictive performance. Therefore, datasets need to be representative of the patient population within which the AI model is designed for. Addressing bias within datasets is imperative, as such bias can propagate into clinical practice, potentially leading to harmful disparities in care. To mitigate this risk, the previous literature has emphasised the importance of evaluating systematic gender and racial biases in AI models [21], as well as tackling the underrepresentation of specific cardiovascular conditions, for instance, congenital heart disease [22].

Despite best efforts, missing data is a common issue which complicates statistical analysis and can lead to result bias and incorrect conclusions being drawn [23]. Thus, it is imperative to understand the extent of the missing data within a dataset prior to AI model development and statistical analysis. Approaches to overcome missing data are numerous and have been discussed previously [23,24,25].

Equally important is the dataset size and the incorporation of sample size calculation. This ensures that datasets are sufficiently large to enable statistical power to be achieved in answering the research objective, alongside ensuring AI model predictions are generalisable to wider patient population [26]. However, in healthcare AI studies, it has been noted that a substantial number of studies do not include a justification of sample size despite there being recommendations from the Transparent Reporting of a Multivariable Prediction Model for Individual Prognosis or Diagnosis (TRIPOD+AI) guideline, [27] the ten principles of Good Machine Learning Practice [28], and the Consolidated Standards of Reporting randomised trials-AI [29].

In supervised learning approaches, datasets require expert annotation to identify anatomical structures, pathologies, and other clinically relevant features. Such annotations are typically performed by experienced echocardiographers, ensuring that the established ground truth for model training is accurate and reliable. Establishing a robust ground truth is essential as the performance of an AI model is inherently dependent on the quality of the data on which it is trained [30]. If the ground truth is inaccurate, inconsistent, or based on subjective interpretation, these errors will propagate into the AI model, leading to unreliable predictions and potentially unsafe clinical decision making [31]. However, there are limitations in obtaining ground truths including the capability of creating large labelled datasets and the ambiguity and imperfect annotations that are used in ground truth labelling [2,32]. For these reasons, multi-expert consensus or validation against established diagnostic tests are increasingly recommended for AI echocardiography research. A compelling example is seen in Howard et al., [13] whereby 26 expert echocardiographers independently labelled the dataset enhancing the reliability of the AI model in assessing left ventricular dimensions, providing a robust benchmark with human-level precision.

### 2.3. Dataset Preprocessing

Once a dataset is constructed, it must undergo cleaning, a process to appropriately prepare, format, and scale the data. This step is crucial for improving model accuracy, efficiency, and generalisation [33]. Without this, an AI model may become too specific to the training dataset and subsequently perform poorly on unseen data, a phenomenon known as overfitting. Preprocessing steps (See Table 1) can be numerous and include identifying and removing errors and duplicated data, handling missing values, handling noise, and ensuring consistency across variables within a dataset. For structured tabular data, this may involve standardisation, normalisation, or categorical encoding to enable fair comparison across variables [34]. For datasets compromising echocardiographic images, standardisation of the images must be undertaken. Here, images are re-sized, normalised, temporally aligned and augmented (for example through rotation or brightness adjustment) to improve generalisation [35]. Annotation quality control is also essential, as noisy or inconsistent labels can significantly degrade model performance [36].

## 3. Training of Echocardiography Artificial Intelligence Models

### 3.1. Training

**Figure 2:**
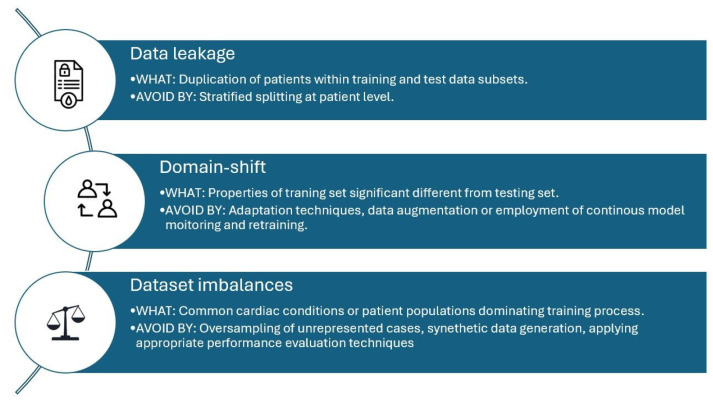
Echocardiography AI training model risks.

Effective evaluation of an AI model typically includes an initial training phase, testing phase and followed by internal and external validation (internal and external validation). As a result, distinct datasets are required, which should be partitioned from each other to ensure independence of data. These datasets are often described as training, testing, and validation sets. This division ensures that the AI model not only learns effectively from the data but also generalises well to previously unseen cases [37]. If a single dataset is used, several data splitting techniques can be employed, including random splitting, stratified splitting, time-based splitting, and cross-validation [38]. Each method has its own advantages and limitations, and the choice of technique should align with the original research objective and the nature of the available data. When considering training sets, there are several key risks that can result in suboptimal AI model performance. These include data leakage, domain-shift, and dataset imbalances; an overview of these and how they can be overcome are shown in Figure 2.

Once the data has been split appropriately, the training phase can begin. In supervised learning, the model iteratively learns from labelled training data by minimising a predefined loss function, which quantifies the difference between the AI model’s predictions and the known ground truth. Optimisation algorithms such as gradient descent are used to update the models parameters (e.g., weights and biases) based on the calculated gradients of the loss function [37]. Training continues until the model converges to a solution that balances performance on both the training and testing/validation sets, with the goal of avoiding underfitting and overfitting [39]. This approach has enabled echocardiography AI models to automatically classify multiple standard views, segment cardiac structures, and even estimate left ventricular ejection fraction [40]. In contrast, unsupervised learning lacks ground truth labels. Here, optimisation often involved minimising reconstruction error or maximising the similarity within clusters. A notable application in echocardiography is the study by Chao et al., [41] who developed a left ventricular diastolic function classification and risk stratification AI model. Here, nine diastolic function parameters were used to identify three distinct diastolic function phenotype clusters (normal diastolic function, impaired relaxation, and in-creased filling pressure), with associated 3-year mortality rates of 11.8%, 19.9%, and 33.4%, respectively.

### 3.2. Development

For AI echocardiography models that interpret images, feature extraction techniques are commonly employed to capture spatial and temporal patterns. Deep learning-based approaches can automate the extraction of these features. As demonstrated by Ouyang et al., [42] convolutional neural networks were used to predict left ventricular ejection fraction from echocardiographic images. This step is particularly important for echocardiography AI models, where image quality limitations can impair both human operator analysis [43] and AI model performance [41]. For structured tabular data, feature selection methods (e.g., wrapping, filtering, and embedding) can identify the most clinically relevant parameters. For example, Samad et al., [44] applied a random forest wrapping method to condense over 400 echocardiographic and clinical variables into the top ten features that were most predictive of all-cause mortality. This enabled the AI model to achieve 95.5% accuracy in predicting 5-year all-cause mortality. Alternatively, a feature reduction technique, known as principal component analysis, can be used which aims to reduce the number of variables needed within AI models whilst preserving the variance seen within a dataset. While these approaches can reduce dimensionality and improve computational efficiency, they often lack transparency, making the results more challenging to interpret in clinical practice [45]. Consequently, healthcare professionals may prefer echocardiographic measurements that are familiar, clinically validated, and evidence based.

### 3.3. Performance Evaluation

Performance evaluation involves systematically examining occurrences for where the AI model underperforms. For instance, Ouyang et al., [42] highlighted that their AI model, which was developed to provide left ventricular ejection fraction estimates, consistently produced larger performance errors when assessing patients with poor acoustic imaging windows and when regional wall motion abnormalities were present [42]. This feedback is crucial in improving the AI model’s performance as it allows for the refinement of the steps within the initial stages of preprocessing or feature extraction. As shown in Table 2, this can be performed using various techniques and is dependent on the specific task the AI model was designed to perform.

In addition to these metrics, an AI model’s performance is often compared with that of human echocardiographers, where the goal is to assess whether the AI system can match, or surpass, the diagnostic accuracy and interpretive capabilities of human echocardiographers. This was highlighted in Upton et al., [9] whereby an AI-based model was designed to interpret stress echocardiograms for the detection of severe coronary artery disease. Here, four human experts assessed the stress echocardiograms, both with and without support from the AI model. The results showed that the AI-supported approach improved the detection rate of coronary artery disease from 85% to 95%, while also increasing diagnostic confidence of the human expert echocardiographers by approximately 10% and reducing the proportion of uncertain diagnoses by 29%.

## 4. Validation of Echocardiography Artificial Intelligence Models

### 4.1. Internal Validation

Internal validation is an important component of AI model development which is aimed at assessing an AI model’s performance and evaluating its predictive accuracy using a subset of data that was initially held back from the dataset that was used in the training and test. This process helps to mitigate the risk of overfitting, improves model robustness, and enables efficient use of data [39]. However, since the same data is used, model performance may be overly simplistic as it often fails to capture variability, which can be seen different patient populations, echocardiography vendors, or hospital settings.

An example of internal validation in echocardiography research is presented in the study by Valsaraj et al., [46] in which an AI model was developed to predict all-cause mortality based on echocardiographic imaging. The dataset included 3626 patients and was divided into five folds for cross-validation. This approach allows an AI model to be trained on four folds and subsequently internally validated on the remaining fifth fold. This technique provided a robust internal estimate of model generalisability within the dataset while leveraging all available data for both training and validation purposes.

### 4.2. External Validation

The next step in the validation process of AI models is external validation, which involves the use of an independently derived dataset to validate the performance of an AI model (see Table 3). This is important for determining the generalisability of an AI model. Additionally, it can be used to provide assurance that an AI model is able to perform well on different patient populations to which the AI model was initially trained and tested on [47]. As discussed in Fletcher et al., [48] the use of external validation with an independent dataset provides confidence in the absence of overfitting. Additionally, underfitting can be detected through external validation, where perhaps the training data was too small and/or included too little variability, resulting in the AI model performing the desired task to a suboptimal accuracy level [47]. However, it has recently been argued that external validation does not guarantee generalisability, and studies have shown that AI models are unreliable when tested on an independent dataset [49]. This has led to growing calls for site-specific validation, which involves conducting reliability testing at each clinical site prior to AI model deployment, followed by ongoing validation checks throughout the model’s lifecycle to ensure continued accuracy and relevance to the local patient population. In echocardiography, concrete strategies to support this include multi-centre prospective validation studies and federated learning frameworks. For instance, a population-scale federated learning study in Germany successfully retrained an automated echocardiographic measurement AI model across 3226 participants, achieving more robust and consistent results than both the original tool and expert readers [50]. Another study externally validated a deep learning algorithm for global longitudinal strain across diverse cohorts, showing strong agreements with manual measurements despite differences in population and imaging protocols [51]. These examples underscore the importance of specific, multi-institutional validation approaches to ensure AI models are safe, accurate, and clinically useful across varied real-world environments.

### 4.3. Clinical Validation

**Figure 3:**
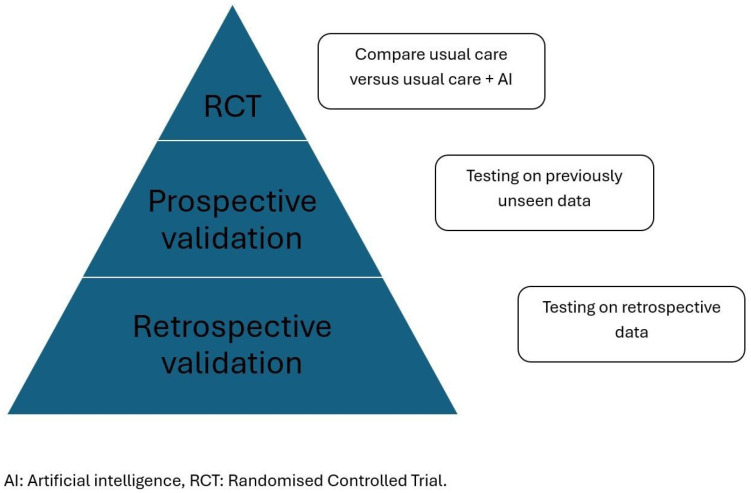
Clinical validation of echocardiography AI models.

Clinical validation involves testing an AI echocardiography model in real-world healthcare settings to assess its utility, accuracy, and safety; this can be achieved in several ways (See Figure 3). However, to date, only a small number of prospective validation and randomised controlled trials (RCTs) have been conducted, suggesting it remains an emerging field [56]. Table 4 highlights prospective validation trial that are currently ongoing.

An example of combined retrospective and prospective validation is shown in Malins et al., [57] where a convolutional neural network AI model was developed to estimate left ventricular ejection fraction from echocardiography images. This study utilised a combination of retrospective datasets alongside a prospective collation of echocardiography images, enabling the model to be trained and initially tested on existing data while immediately assessing its performance on new, real-world patient data, which helps identify potential biases, ensures generalisability, and may accelerate the evaluation of clinical utility. An example of an RCT is shown in Narang et al., [7] where authors evaluated a deep learning AI model that provided real-time guidance to novice operators in the acquisition of 10 echocardiography images. Here, eight nurses with no prior experience used the AI tool to scan 240 patients achieving diagnostic quality images in over 98% of cases for key measurement including left ventricular size and function, right ventricular size, and pericardial effusion. The findings show that AI guidance can enable non-experts to reliably acquire limited diagnostic echocardiography images, potentially expanding access to cardiac imaging in resource-limited or remote settings.

While many AI models demonstrate high performance in retrospective and prospective studies, these findings often rely on curated datasets and may not be generalisable to the wider patient populations or clinical workflows [58,59]. However, RCTs provide a robust framework to assess whether an AI model is able to improve patient outcomes, clinician decision making, workflow efficiency, or diagnostic accuracy compared with standard care [29]. They are also crucial for uncovering unintended consequences, such as automation bias or differential performance across demographic subgroups [52]. An example of a RCT involving an AI echocardiography model is PROTEUS, a prospective study that evaluated the use of AI in stress echocardiography [52]. Here, Upton et al., evaluated whether AI-augmented decision making (intervention) was non-inferior to standard decision making (control) when selecting patients for invasive coronary angiography following stress echocardiography. The results showed that among the 2341 patients in the study, the sensitivity and specificity of the intervention and control groups were similar, meaning both methods performed comparably in identifying true positives and true negatives. Yet despite this, a sub-analysis suggested that the intervention might offer some benefit to low volume stress echocardiography services.

### 4.4. Regulatory and Medical Device Approval

In the United States, the Food and Drug Administration (FDA) requires retrospective and prospective evidence that AI models are safe, effective, and generalisable before market clearance as a medical device is provided. A similar approach is in place in Europe (e.g., medical device regulation) and the UK (e.g., Medicines and Healthcare products Regulatory Agency). However, the regulatory approval process of AI models within healthcare continues to evolve with the FDA recently releasing guidance for their initial evaluation and approval [60].

For echocardiography AI models, where measurements such as left ventricular ejection fraction or valve function directly guide management decisions, RCTs remain the gold standard to demonstrate clinical validity and support ethical deployment into routine use [61]. While this rigorous approach provides strong evidence of safety and clinical benefit, it comes with compromises. The benefits include robust validation in real-world settings, stronger clinician and patient trust, and clearer justification for regulatory approval. Nevertheless, the disadvantages include the high cost and time required to conduct RCTs [62], potential limitations in capturing patient population diversity [63], and the risk that smaller developers may face barriers due to stringent evidence requirements.

### 4.5. Post Marketing Surveillance

Post-market surveillance is vital in detecting model performance degradation (e.g., domain drift, new risks, or reduced accuracy) in echocardiography AI models once deployed. As such, regulatory bodies emphasise continuous monitoring and clinical follow-up to maintain safety and effectiveness in real-world practice. The FDA’s medical device reporting system is one such post marketing surveillance tool that can be used for the ongoing management of AI model performance [64]. However, studies show that only a small fraction of AI models include formal surveillance plans. This was shown in Dolin et al., [65] where only approximately 9% of all FDA approved healthcare AI models included plans for post-deployment surveillance. This strikingly low prevalence underscores the need for more rigorous and proactive strategies to allow the early detection of issues and support the iterative improvement in AI model performance without compromising patient safety.

## 5. Future Considerations

A growing number of guidelines have been developed to enhance transparency, reproducibility, and methodological rigour in AI medical imaging research, addressing concerns regarding the ‘black box’ nature of some models. A recent review identified 26 AI-related health research guidelines published between 2009 and 2023, covering general AI research, field-specific reporting, and guidance for different research phases (pre-clinical, translational, clinical) [66]. Despite available guidance for developing and reporting AI in health research, adherence is inconsistent, limiting the clinical application of many published studies. Furthermore, many AI research studies are published on preprint servers and thus, do not undergo the peer review process [67].

Common issues include incomplete dataset descriptions, unreported dataset bias, and lack of external validation, with fewer than 20% of AI imaging studies including this [58,66]. Broader adoption of these guidelines is crucial for improving the quality and impact of AI models in echocardiography and healthcare. Consequently, journals increasingly require or encourage adherence to AI-specific reporting standards [68].

The exponential growth in research into AI models aimed at addressing unmet clinical needs in echocardiography has yet to be effectively and responsibly translated into clinical practice [69]. One of the primary barriers to implementation is the technical challenge posed by heterogeneous information technology (IT) infrastructures across healthcare systems. While bespoke IT systems allow individual institutions the flexibility to tailor solutions to their specific needs, this often leads to a lack of standardisation and incompatible data formats. These inconsistencies can significantly hinder an AI model’s ability to perform its intended tasks accurately. Another critical consideration is data security. For many healthcare providers, particularly those with strict regulatory requirements, AI models must function entirely within the existing IT infrastructure without transmitting data externally. To address this, AI models are increasingly being developed to be vendor neutral. Studies have demonstrated that such models can be integrated into high-volume echocardiography centres within as little as four weeks [70]. Additionally, compatibility with picture archiving and communication systems (PACSs) is essential. PACS typically utilises the DICOM (digital imaging and communications in medicine) standard for managing, storing, and transmitting medical imaging data. In high-income countries, the adoption of DICOM-compatible PACS is nearly universal. However, this is not always the case in low- and middle-income countries, where access to such infrastructure can be limited [71]. Furthermore, there are inconsistent DICOM encoding between vendors which can create fragmented and inaccessible data [18]. To address this disparity and support the integration of AI into clinical workflows, the development of open, interoperable platforms is critical. These platforms must enable seamless data exchange between AI models, PACS, and other imaging equipment, thereby avoiding data silos and facilitating efficient clinical implementation.

There are significant ethical and liability concerns surrounding the implementation of AI in echocardiography and healthcare in general. It has been argued that a structured four-step process (comprising data acquisition, AI model development, validation and testing, and finally dissemination) is essential for the responsible deployment of AI technologies [68,72]. This framework not only supports the technical robustness of AI systems but also fosters clinical and patient trust. Moreover, early and meaningful stakeholder engagement, including the involvement of patients and public members, is critical during the initial stages of AI model problem definition. This collaborative approach ensures that AI is applied appropriately by assessing, in advance, the utility, feasibility, data availability, cost, deployment challenges, clinical uptake, and long-term maintenance requirements [73].

Echocardiography machines are starting to see the initial integration of AI technologies with several echocardiography cart vendors incorporating streamlined workflows with automated image view recognition, measurement, and Doppler analysis. These have shown promise in the reduction in ultrasound keystrokes and scan time without a reduction in image quality and interpretability. This may lead to improved working ergonomics compared with current echocardiography practice [74]. The application of AI is also extending into three-dimensional (3D) echocardiography, which presents unique challenges due to the complexity and volume of data acquired. Recent developments have shown that AI can assist in automating 3D image segmentation and volumetric quantification [75], significantly enhancing the reproducibility and efficiency of 3D echocardiographic analysis. These AI models may help overcome existing barriers to widespread 3D echocardiography adoption, such as operator variability and long postprocessing times.

In the short term, echocardiography services may benefit from AI solutions developed by commercial vendors that have received regulatory approval for specific clinical applications. For example, AI models have been approved for the assessment of ischaemic heart disease [52], heart failure with preserved ejection fraction [10], aortic stenosis [76], cardiac amyloidosis [77], and cardiac chamber measurement analysis [13]. These models offer the potential to enhance diagnostic efficiency and accuracy. However, the extent to which they become fully integrated into routine echocardiography workflows will ultimately depend on several factors, including implementation time, associated costs, and alignment with specific clinical needs.

In the longer term, the integration of large language models in echocardiography imaging platforms may assist with automated echocardiography reporting, generating structured reports directly from data obtained from the echocardiogram. This may reduce the reporting time and, more importantly, reduce reporting variability [73]. Combined with multimodal AI models that are capable of processing echocardiography images, tabular variables, and text, these systems could interpret echocardiographic findings in the clinical context, linking measurements to patient history, laboratory values, and clinical guidelines for a more personalised approach to patient care [78]. Generative models may also enable real-time educational feedback during image acquisition and interpretation, improving training and reducing inter-operator variability [61]. Yet despite these promising applications, challenges in echocardiography accuracy, AI algorithm explainability, data privacy, and the ethical use of AI in healthcare remain unresolved and thus need addressing prior to the widespread clinical deployment of AI.

## 6. Conclusions

Echocardiography is a pivotal non-invasive imaging modality for the assessment of the hearts structure and function. Interest in integrating AI into echocardiography is rapidly increasing, with its future applications poised to fundamentally transform clinical workflows. This narrative review outlines the pathway typically followed for developing and validating AI models in echocardiography including the key components and challenges faced in these processes, as well as the future considerations for the practical application of AI models within echocardiography.

## Figures and Tables

**Table 1:** Data preprocessing steps.

Preprocessing Stage	Summary	Considerations/Challenges
De-identification	Patient identifiable data needs to be removed prior to analysis, whether this entails patient identification on an image or patient-specific structures/anatomy that could lead to their identification.	Manually removing this data can be time-consuming; automatic processes need to be verified to appropriate data protection standards. Essential anatomical data may be unintentionally removed.
Missing data	Occurrence of missing data (images or tabular data) from the dataset. This can occur due to data not being recorded, data being unavailable, or due to the removal of known errors or outliers.	Missing data can introduce bias as valuable data may be missing. This is especially the case when data is missing, not at random. There is a trade-off between a cleaned dataset and the preservation of the data to allow for appropriate model performance. Computation imputations can assist with missing data.
Format standardisation	Typically, echocardiographic images used in clinical practice are acquired in DICOM format; however, images may need to be converted to a simpler file format to allow adequate analysis.	Available data conversion tools can allow for visual inspection of data from the user during standardisation, allowing image inconsistencies to be identified before analysis.
Noise reduction	Removal of artefactual image noise, such as with a low-pass filter or through data smoothing to enhance image quality.	Risk of unintentionally filtering true cardiac structures if inappropriate filters are applied.
Normalisation	Ensuring a scalable relationship of frames per second (FPS) on echocardiographic images to allow for consistent image analysis.	Lower FPS values than desired could introduce accidental data bias if inappropriately augmented; conversely the removal of frames from higher FPS images could introduce asynchronous image analysis.

**Table 2:** Performance evaluation metrics in the development of AI models.

Performance Metric	Definition	Common AI Model Applications
Accuracy	Percentage of correct prediction made by an AI model. For instance, the diagnostic accuracy of contrast echocardiography for detecting left ventricular thrombus.	Classification and diagnostic models
Sensitivity	True positive rate, how well an AI model detects a positive case. For instance, detecting the presence of aortic stenosis when this is truly present.	Disease detection and screening classification tasks
Specificity	True negative rates, how well an AI model identifies normal cases correctly. For instance, patients without obstructive ischemic heart disease having a normal stress echocardiogram.	Rule-out classification tasks
AUC-ROC	How well an AI model can distinguish between different classes. For instance, distinguishing between patients who have severe left ventricular systolic impairment, impaired left ventricular systolic function, and normal left ventricular systolic function.	Binary and multi-class classification tasks
Precision and Recall	How many of the AI model’s positive predictions are correct (precision) and how many positive cases are identified by the AI model (recall).	Imbalanced classification tasks

AUC-ROC: Area under the receiver operating characteristic curve.

**Table 3:** Examples of external validation in echocardiography AI models.

Study/AI Model/Author	AI Task	Internal Training Dataset	External Validation Dataset	Performance
PROTEUS [9,52]	Detection of coronary artery disease on stress echocardiography and appropriate referral to invasive coronary angiography	EVAREST, multiple UK NHS Trusts (NCT03674255).	Rainier study (Oregon Health Science University Study, Portland, OR, USA.	Internal training dataset: AUROC: 0.934. Specificity: 85.7% (95% CI: 82.7, 88.9%). Sensitivity of 86.7% (95% CI: 80.2, 94.3%) External validation dataset: AUROC of 0.927. Specificity of 92.7% (95% CI: 87.8, 97.6%). Sensitivity of 84.4% (95% CI: 73.9, 95.0%).
EchoNet-Dynamic [42]	LVEF	EchoNet-Dynamic (Stanford University)	Cedars-Sinai Medical Centre	AUROC 0.97 for classifying LVEF thresholds of <40% and >60% in internal training and external validation dataset.
ML algorithm to Automate Morphological and Functional Assessments in 2D Echocardiography [53]	HCM vs. athlete’s heart classification	Mount Sinai Hospital (New York)	Independent external cohort	AUROC of >0.93 in internal training and external validation dataset.
EchoGo Heart Failure [10]	Classification of HFpEF, no HFpEF, or non-diagnostic.	Mayo Clinic, Minnesota, US and St Georges Hospital, London, UK.	Mayo Clinic, Minnesota US (patients from a geographically distinct area than those who were included in the internal training set).	Internal training dataset: AUROC: 0.97 (95% CI: 0.96, 0.97). External validation dataset: AUROC: 0.95 (95% CI: 0.93, 0.96).
DL algorithm for automated global longitudinal strain [51]	Automated global longitudinal strain	Mackay Memorial Hospital, Taipei	Prospective multi-national observation study PROMISE-HFpEF [54]	Internal training dataset: Automated measurements showed good agreement versus manual measurements: −18.9 ± 4.5% vs. −18.2 ± 4.4%, bias 0.68 ± 2.52%, MAD 2.0 ± 1.67, RMSE = 2.61, R = 0.84. External training dataset: Automated measurements showed good agreement versus manual measurements: −15.4 ± 4.1% vs. −15.9 ± 3.6%, bias −0.65 ± 2.71%, MAD 2.19 ± 1.71, RMSE = 2.78, R = 0.76.
DELINEATE-regurgitation study [55]	Deep learning algorithm for the assessment and risk stratification of aortic, mitral, and tricuspid regurgitation.			Internal training dataset: Weighted kappa for regurgitation classification: Aortic regurgitation: 0.81 Mitral regurgitation: 0.76 Tricuspid regurgitation: 0.73 External training dataset: Weighted kappa for regurgitation classification: Aortic regurgitation: 0.76 Mitral regurgitation: 0.72 Tricuspid regurgitation: 0.64.

AUROC: Area under the receiver operating characteristic curve, EVAREST: echocardiography value and accuracy at rest and stress, HFpEF: heart failure with preserved ejection fraction, LVEF: left ventricular ejection fraction, MAD: mean absolute difference, ML: machine learning, RMSE: root-mean-squared-error.

**Table 4:** Examples of prospective validation trials for echocardiography AI models.

Prospective Trial	Study Description	Estimated Patient Enrolment	Estimated Year of Study Completion
AGILE-ECHO: Use of artificial intelligence-guided echocardiography to assist cardiovascular patient management. (NCT05558605)	Evaluate effectiveness of AI-guided echocardiography acquisition for triage and management of patients with suspected heart failure and valvular heart disease in rural and remote Australia	612	2025
MAIQUEE: A multi-centre study on artificial intelligence-based quantitative evaluation of echocardiography. (NCT07133516)	Comparison of echocardiographic analysis between automatic AI-performed measurements with manual measurements from physicians of varying experience levels	1600	2025
EchoNet-Screening: Artificial intelligence-guided echocardiographic screening of rare diseases. (NCT05139797)	Evaluation of the EchoNet-LVH algorithm to accurately detect cardiac hypertrophy and identify patients that require additional screening for cardiac amyloidosis	300	2027
AISEARHF: Artificial intelligence versus sonographer echocardiogram analysis and reporting in patients with heart failure. (NCT07021599)	Multicentre RCT comparing echocardiographic analysis between AV versus experienced sonographers	514	2028
AI-SEE: Artificial intelligence stress echo (sub-study of stress echo 2030) (NCT05081115)	AI-SEE images: Operator-independent image interpretation of stress echocardiography, including assessment of coronary flow reserve, diastolic function, and right ventricular function AI-SEE data: Use of DL algorithms to create personalised patient risk prediction models.	10,000	2030

## Data Availability

No new data were created or analysed in this study. Data sharing is not applicable to this article.

## Abbreviations

AI	Artificial intelligence
DICOM	Digital Imaging and Communications in Medicine
FDA	Food and Drug Administration
IT	Information Technology
ML	Machine Learning
NCT	National Trial Number
RCT	Randomised Controlled Trial
PACS	Picture Archiving and Communication Systems
